# Fatal Attraction Phenomenon in Humans – Cat Odour Attractiveness Increased for *Toxoplasma*-Infected Men While Decreased for Infected Women

**DOI:** 10.1371/journal.pntd.0001389

**Published:** 2011-11-08

**Authors:** Jaroslav Flegr, Pavlína Lenochová, Zdeněk Hodný, Marta Vondrová

**Affiliations:** 1 Department of Biology, Faculty of Science, Charles University, Prague, Czech Republic; 2 Department of Anthropology, Faculty of Humanities, Charles University, Prague, Czech Republic; 3 Department of Genome Integrity, Institute of Molecular Genetics ASCR, v.v.i, Prague, Czech Republic; Imperial College London, United Kingdom

## Abstract

**Background:**

Latent toxoplasmosis, a lifelong infection with the protozoan *Toxoplasma gondii*, has cumulative effects on the behaviour of hosts, including humans. The most impressive effect of toxoplasmosis is the “fatal attraction phenomenon,” the conversion of innate fear of cat odour into attraction to cat odour in infected rodents. While most behavioural effects of toxoplasmosis were confirmed also in humans, neither the fatal attraction phenomenon nor any toxoplasmosis-associated changes in olfactory functions have been searched for in them.

**Principal Findings:**

Thirty-four *Toxoplasma*-infected and 134 noninfected students rated the odour of urine samples from cat, horse, tiger, brown hyena and dog for intensity and pleasantness. The raters were blind to their infection status and identity of the samples. No signs of changed sensitivity of olfaction were observed. However, we found a strong, gender dependent effect of toxoplasmosis on the pleasantness attributed to cat urine odour (p = 0.0025). Infected men rated this odour as more pleasant than did the noninfected men, while infected women rated the same odour as less pleasant than did noninfected women. Toxoplasmosis did not affect how subjects rated the pleasantness of any other animal species' urine odour; however, a non-significant trend in the same directions was observed for hyena urine.

**Conclusions:**

The absence of the effects of toxoplasmosis on the odour pleasantness score attributed to large cats would suggest that the amino acid felinine could be responsible for the fatal attraction phenomenon. Our results also raise the possibility that the odour-specific threshold deficits observed in schizophrenia patients could be caused by increased prevalence of *Toxoplasma*-infected subjects in this population rather than by schizophrenia itself. The trend observed with the hyena urine sample suggests that this carnivore, and other representatives of the Feliformia suborder, should be studied for their possible role as definitive hosts in the life cycle of *Toxoplasma*.

## Author Summary

Latent toxoplasmosis, a lifelong infection with the protozoan *Toxoplasma gondii*, has cumulative effects on the behaviour of hosts, including humans. The most impressive effect of toxoplasmosis is the so-called “fatal attraction phenomenon,” the conversion of innate fear of odour of the definitive host, the cat, into attraction to cat odour in rodents infected with *Toxoplasma*. While most behavioural effects of *Toxoplasma* infection were confirmed also in humans, neither the fatal attraction phenomenon nor any toxoplasmosis-induced changes in olfactory functions have been searched for in them. Our study performed on 34 *Toxoplasma*-infected and 134 noninfected students showed that the infected men rated odour of cat urine as more pleasant than did the noninfected men, while infected women rated the same odour as less pleasant than did noninfected women. No significant effect of toxoplasmosis on the urine odour pleasantness was found for horse, tiger, brown hyena and dog. The possible absence of the effects of toxoplasmosis on the urine odour pleasantness score attributed to tiger would suggest that the amino acid felinine, which is absent in urine of large cats, could be responsible for the fatal attraction phenomenon.

## Introduction

The protozoan parasite *Toxoplasma gondii* is well known for changing behaviour of intermediate hosts, for review see [1], [2]. In general, it is believed that specific behavioural changes represent a biological adaptation of the parasite aimed to enhance its transmission from an intermediate host (e.g. a rodent) to a definitive host (any cat species) by predation. Consequently, the infected rodents have prolonged reaction times [3], increased (rats) or decreased (mice) preference for novel stimuli [4]–[7], impaired learning ability [6], [8], [9], and increased activity [10], [11] including spontaneous activity in running wheel tests [6], [12]. Additionally, infected rats have a higher probability to be captured with traps [4], which is consistent with the results of the “predation experiments” performed with other taxonomically related parasites with similar life cycles (*Sarcocystis* and *Frenkelia*) [13].

The most impressive effect of *Toxoplasma* infection on the behaviour of rodent hosts is the so-called “fatal attraction phenomenon” [14]. In contrast with *Toxoplasma*-free rodents that have an innate fear of cat odour and avoid the places containing traces of cat urine, the *Toxoplasma* infected animals, both mice and rats, lose this fear and even gain attraction to cat odour instead. In the arena experiments, these animals spend more time in places containing a cat urine sample or piece of a worn cat collar. This effect is highly specific, the fatal attraction concerns only the odour of cat or bobcat and not of a rabbit, mink or dog [15]–[17]. The general qualities of olfaction as well as other fear or anxiety reactions of the infected animals remain unchanged [15]. Moreover, this specific effect of the *Toxoplasma* infection disappears when the infected animals are treated with drugs that specifically inhibit proliferation of *Toxoplasma* [17], [18]. In contrast with many other *Toxoplasma* infection-induced behavioural changes, the fatal attraction phenomenon is rather stable and remains detectable for a longer period of time than is the average life expectancy of rodents in their natural environment [19].

*Toxoplasma* infects any warm-blooded animal, including about one third of the human population of developed countries [20]. Thus, humans are very suitable models for studying possible manipulation activity of the *Toxoplasma*. Acute toxoplasmosis is a relatively mild and short-term disease in most of the immunocompetent subjects. Acute toxoplasmosis goes spontaneously into the latent phase, characterized by the lifelong presence of dormant stages of the parasite, the bradyzoites, mainly in the neural and muscular tissues, usually also by the lifelong presence of low but protective concentrations of specific antibodies in the serum of infected subjects [21]. Any behavioural changes that could be detected several years after the infection are either side-effects of latent infection or specific effects of manipulation activity of the parasite rather than carry over effects of the past acute phase of the infection [2]. This theoretical assumption was supported by evidence of positive correlation between the intensity of many behavioural changes and duration of the infection [22]–[25].

It was observed that infected humans have prolonged reaction times, increased risk of traffic accidents – analogy of the “predation accidents” that are studied in predation experiments in animals [26], [27], changed personality profile, namely a decreased Novelty seeking score in Cloninger's TCI test [28], [29], changed superego strength (increased in women and decreased in men), protension (decreased suspiciousness in women and increased in men) and affectothymia (increased warmth in women and decreased or unchanged in men) in Cattell's 16PF questionnaire [30], [31]. The infected humans also differ in behaviour in normal situations, in artificial situations of behavioural experiments, in clothing tidiness (increased in women and decreased in men), and express different economic behaviour in experimental games (increased altruism in women and decreased altruism in men) [32], [33]. The infected subjects have changed levels of testosterone (decreased in women and increased in men). There is also indirect evidence for increased levels of dopamine in the brain of infected subjects [28], [29], which could explain the causation of the observed association between toxoplasmosis and schizophrenia [34]–[36], autism [37], Parkinson's disease [38], [39] and Alzheimer disease [40]. Several recent studies showed increased risk of suicides in the infected subjects [41], [42].

To the authors knowledge, no results concerning the effect of latent toxoplasmosis on human olfactory function have been published yet. However, it was shown recently that schizophrenic patients who are reportedly more often infected with *Toxoplasma* than the general population [35] express odour-specific threshold deficits [43] possibly related to a disruption of cAMP-mediated signal transduction [44]. The fatal attraction phenomenon has not been studied in any animal species other than mice and rats. In the present study we: 1. searched for any evidence of toxoplasmosis-associated quantitative or qualitative changes in human olfactory functions; 2. searched for the existence of the fatal attraction phenomenon, i.e. the increased attractiveness of cat urine odour to humans. Our population of *Toxoplasma*-infected and *Toxoplasma*-free university students were asked to rate the odour of urine samples from five animals, the cat, horse, tiger, brown hyena, and dog, for the intensity and pleasantness. The raters were blind to the *Toxoplasma* infection status and identity of the samples rated.

## Methods

### Ethics statement

The project and specific subprojects were approved by the IRB of the Faculty of Science, Charles University. All participants signed a general informed consent form to participate in a toxoplasmosis study before the blood test and all subsequent evaluations. The samples of animal urine for testing olfactory ability and preferences of students were obtained from spontaneously urinating animals without any contact with the animals, therefore, according to international and national guidelines and commendations of the IACUC of Faculty of Science, Charles University, no formal approval of this part of experimental protocol by an IACUC or an ethic committee was necessary.

### Subjects

The students of the Faculty of Science of Charles University were invited, during regular baccalaureate courses of biology, to participate voluntarily in an unspecified study aimed to search for phenotypic, mostly behavioural effects of latent toxoplasmosis. More than 50% of attendants of these courses participated in the first testing session and provided a blood sample for serological testing for toxoplasmosis and also an e-mail address and mobile phone number. Later, they were invited by e-mail or text message for half-day testing sessions, including the test of olfactory preferences. Some of these sessions (e.g. experimental game session and olfactory abilities session) were remunerated, while others were not (e.g. reaction time and intelligence testing sessions). The students received 200 CZK (about $ 10) for their participation in the olfactory functions experiment.

### Origin of urine samples

To gather all target urine samples, we solicited a veterinary surgery (cats and dogs), the Prague zoo (horses, tigers, and brown hyenas) and the second author's friends (horses and dogs). The collaborators were instructed to collect clear urine preferably directly from animals while urinating, if possible. Immediately after collection, the samples were aliquoted into 1 ml plastic tubes (to facilitate handling) and subsequently deep frozen (−72°C) until used in the testing sessions.

The available urine samples from various animal species (altogether 5 cats, 5 hyenas, 5 tigers, 3 dogs, and 3 horses) were used as specified below and carefully randomized. Thus, in each session, a specific counterbalanced set of 10 vials from different individuals (2 vials per species) were evaluated. Based on our previous experience and on the fact that the effect of toxoplasmosis on olfactory preferences qualitatively differs depending on the intensity of cat odour [16], we decided to test two samples of urine from each species, differing in the dose and hence most likely in the odour intensity (a specific amount of each sample was assessed, as derived from a pilot research project conducted approximately one year prior to this study and help with finalizing the experimental design). Specifically, the samples were dosed as follows: 50 µl and 100 µl of horse and cat urine and 20 µl and 40 µl of tiger, hyena, and dog urine.

### Testing of olfactory ability and preferences

The experiment consisted of 17 rating sessions. Each session started in the morning (after the samples had been thawed for 1.5 h and prepared for testing) and continued to approximately 2 pm. It took place in a small but convenient experimental room at a stable room temperature and humidity (average temperature ranged from 20.6 to 25°C and average humidity from 42.75 to 56.6%).

A specific amount of each sample was applied on a cotton pad (100% cotton, elliptical in shape, approximately 7 cm at the longest diameter; Ebelin cosmetic pads, DM-drogeriemarkt, Ceske Budejovice, Czech Republic) that was subsequently placed in a 500-ml opaque laboratory jar with a glass lid and labeled with an alphanumeric code that changed with each session. The samples used for a session were divided into 2 identical sets, with the less and more intense odour samples evenly distributed. After sniffing half of the urine samples, the raters were recommended to have at least a 5-minute break to avoid possible odour habituation. During the break, they were encouraged to drink water and smell coffee beans for olfactory recovery.

The stimuli were assessed on 7-point scales for the pleasantness and intensity. The two ends of each scale were anchored by the verbal descriptors (e.g., very unpleasant and very pleasant). The rating scores were written down immediately after sniffing the respective stimulus and the time spent by sniffing was not restricted. If the raters found any of the samples too weak, they were instructed to select the answer “I cannot smell the sample” instead of using the scales.

### Testing for toxoplasmosis

The sera of students were tested for the presence of anamnestic concentrations of anti-*Toxoplasma* antibodies with IgG ELISA (SEVAC, Prague) in the National Reference Laboratory for Toxoplasmosis of the Czech Republic. The subjects with very high concentrations of antibodies were tested for acute toxoplasmosis with IgM ELISA (SEVAC, Prague). Only the subjects with positive result of IgG ELISA (concentration of specific antibodies>10 IU/ml) and negative in IgM ELISA (positivity index less than 0.9) were considered to be latent toxoplasmosis positive and were included in the present study. One female student with acute phase toxoplasmosis was informed about the serological test result and was not used for any part of our experiment.

### Statistical analyses

All statistical testing, including analyses for the tests assumptions, namely those of the normality of data distribution, normality of residuals and equality of error variances, was performed with Statistica 9.0. The differences in the attributed intensity and pleasantness scores were estimated with the General Linear Models method (GLM), using the intensity or Z-score of the odour pleasantness scores attributed to a particular sample as dependent variables and the rater's sex and toxoplasmosis status as independent factors. To eliminate any potential effect of a strong negative correlation between the odour intensity and pleasantness, the odour intensity of a particular sample was included into the models as a covariate. However, when not included, the results were qualitatively the same. Although there was only a little inter-individual variability in the odour intensity rating scores, the study subjects differed in the odour pleasantness scores – some only used the lower part of the 7-point scale while others used the whole range of the scale. To control for individual rating habits, we used the Z-scores instead of raw ratings (numbers 1–7) for the odour pleasantness assessment. The Z-score was obtained by dividing the difference between the subject's rating score for a particular sample and mean rating score for all samples by the standard deviation of all his/her rating scores.

## Results

**Figure 1:**
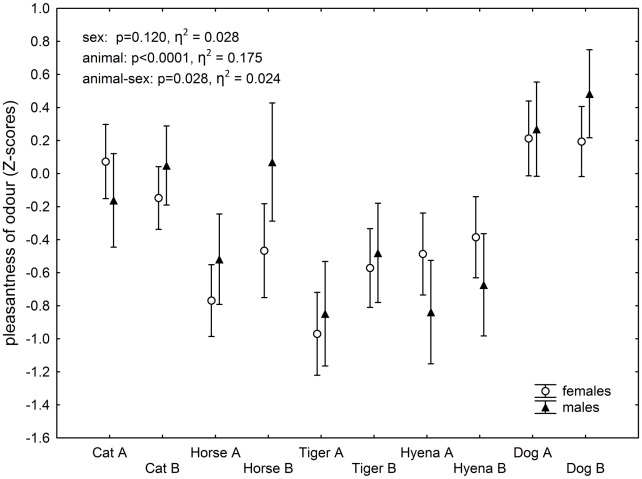
Differences in the urine odour pleasantness scores attributed to various species of animals. The empty circles and triangles denote mean Z-scores for women and men, respectively; the vertical bars denote 0.95 confidence intervals. The odour of samples with more positive Z-scores was scored as more pleasant (in comparison with other urine samples). The means were controlled for intensity of the odour attributed by particular rater, i.e. they were computed for mean intensity of the odour. A and B are the high and low concentration samples, respectively (see the Methods section).

**Figure 2:**
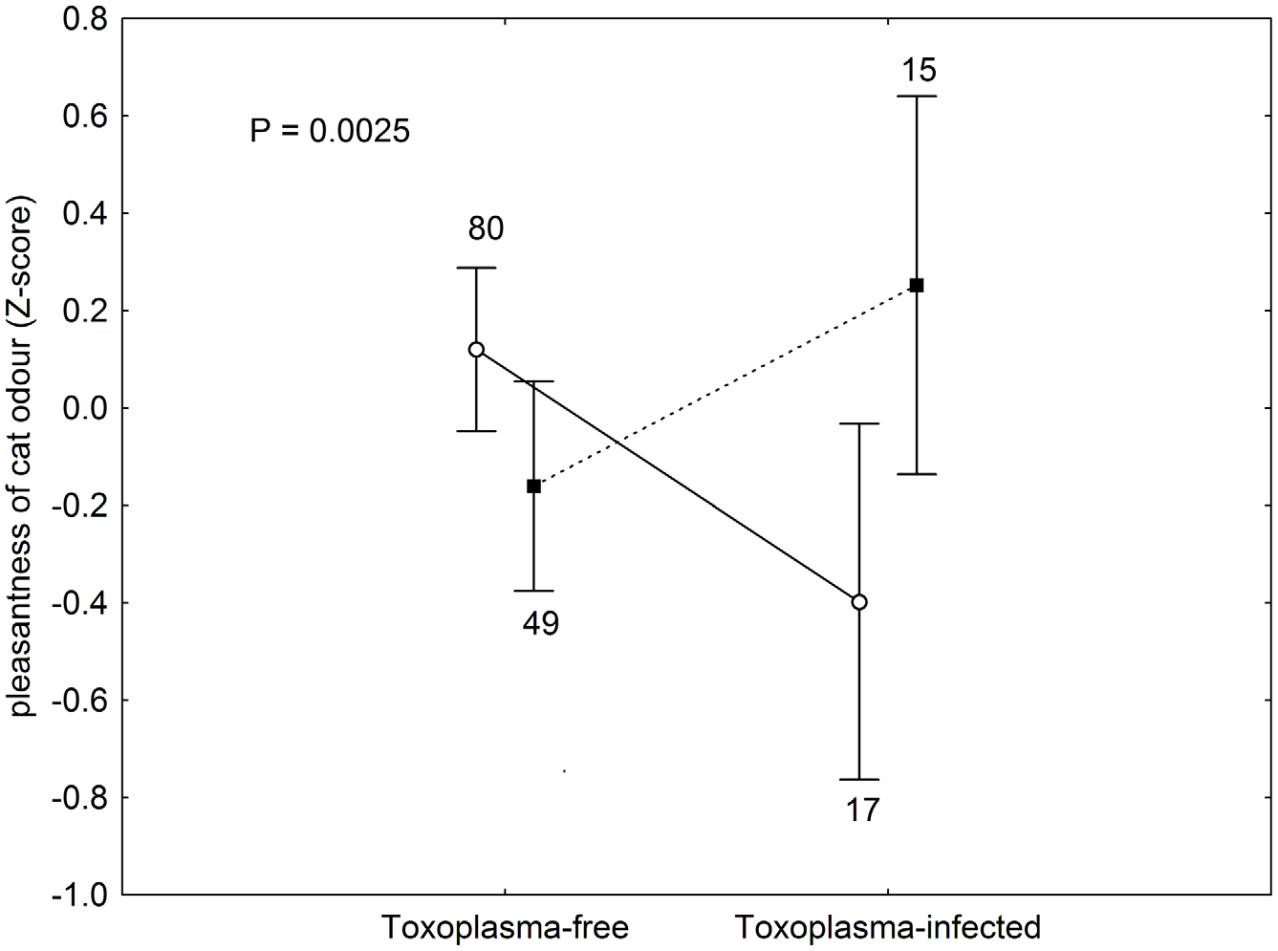
The cat urine odour pleasantness. The figure shows odour pleasantness scores attributed to the cat urine sample by *Toxoplasma*-infected and *Toxoplasma*-free male and female students. The circles and squares denote mean Z-scores for women and men, respectively; the vertical bars denote 0.95 confidence intervals. The odour of samples with more positive Z-scores was scored as more pleasant (in comparison with other urine samples). In contrast to results presented in Tab. 1, here the means were controlled for intensity of the odour attributed by particular rater, i.e. they were computed for mean intensity of the odour.

The final set contained data on 102 women (19 *Toxoplasma*-infected) and 66 men (15 *Toxoplasma*-infected). The difference in the frequency of toxoplasmosis between women and men was not significant (Chi^2^ = 0.417, p = 0.518). There were no sex differences in the mean odour pleasantness and intensity scores for all 10 samples (pleasantness: t = −1.139, p = 0.167; intensity: t = −0.079, p = 0.937). The urine odour pleasantness scores varied with the animal species, see Fig. 1. Because of low concentration of urine samples and a probable odour-specific hyposmia of particular raters, only about half of the study subjects were able to detect and to rate all ten samples. Therefore, we did not use the multivariate test for all specimens and analysed the odour pleasantness of the particular samples in separate GLM tests, with the Z-score of the odour pleasantness of a sample as a dependent variable and the rater's sex (male-female) and toxoplasmosis status (infected-noninfected) as two binary factors. The odour pleasantness always correlated negatively with the odour intensity. Therefore, we included the attributed odour intensity score in the model as an ordinal covariate. The results of these GLM analyses are shown in Table 1. Of all 10 samples, only the more concentrated cat urine sample showed a significant effect of the toxoplasmosis status, namely a highly significant effect of the toxoplasmosis-sex interaction. The infected men rated cat urine odour as more pleasant than did the *Toxoplasma*-free men, while *Toxoplasma*-infected women rated it as less pleasant than did *Toxoplasma*-free women; a non-significant trend in the same directions was observed also for hyena (p = 0.074, η^2^ = 0.023). This effect for the cat odour was also significant when a simpler GLM model without any covariate was analysed; however, the p value for the interaction increased from 0.0025 to 0.0043. The observed toxoplasmosis-sex interaction remained significant even after Bonferroni correction for multiple tests. Separate analyses showed that the effect of toxoplasmosis was significant both for women (p = 0.041, η^2^ = 0.044) and men (p = 0.012, η^2^ = 0.098), see Fig. 2.

**Table 1:** Differences in attributed pleasantness and intensity of the smell of urine of different animals.

	women N		men N		Z-score women		Z-score men		effect toxo		effect sex		effect toxo-sex		effect intensity	
	toxo−	toxo+	toxo−	toxo+	toxo−	toxo+	toxo−	toxo+	p	η^2^	p	η^2^	p	η^2^	p	η^2^
cat A	80	17	49	15	0.126	−0.361	−0.182	0.250	0.730	0.001	0.225	0.009	0.002*	0.057	0.000*	0.178
cat B	73	15	48	13	−0.178	−0.309	−0.035	0.001	0.888	0.000	0.111	0.018	0.494	0.003	0.000*	0.133
horse A	83	19	51	14	−0.703	−0.426	−0.483	−0.731	0.633	0.001	0.546	0.002	0.192	0.011	0.001*	0.086
horse B	82	19	49	15	−0.381	−0.206	−0.096	−0.278	0.754	0.001	0.896	0.000	0.214	0.010	0.000*	0.147
tigr A	83	19	50	15	−0.825	−1.028	−0.762	−0.943	0.455	0.003	0.659	0.001	0.873	0.000	0.000*	0.129
tigr B	80	19	51	15	−0.543	−0.568	−0.449	−0.615	0.747	0.001	0.704	0.001	0.832	0.000	0.000*	0.205
hyena A	73	15	43	14	−0.367	−0.023	−0.722	−0.954	0.949	0.000	0.002*	0.064	**0.074**	0.023	0.000*	0.286
hyena B	68	14	43	14	−0.222	−0.485	−0.473	−0.213	0.754	0.001	0.685	0.001	0.295	0.008	0.000*	0.355
dog A	70	15	46	14	0.197	0.074	0.272	0.107	0.308	0.007	0.961	0.000	0.392	0.005	0.000*	0.120
dog B	64	16	30	14	0.210	0.292	0.558	0.271	0.336	0.008	0.332	0.008	0.335	0.008	**0.061**	0.029

The samples labeled “A“ and “B” represent the high dose and low dose samples, respectively. The effects size are described with η^2^, significant effects (p<0.01) and trends (p<0.1] computed with GLM test are denoted with asterisk and printed in bold, respectively.

There was no effect of toxoplasmosis, sex or toxoplasmosis-sex interaction on the odour intensity scores attributed to any of ten samples, except the more concentrated dog urine (toxoplasmosis-sex: p = 0.047, η^2^ = 0.027); however, this effect was not significant after Bonferroni correction for multiple tests.

## Discussion

*Toxoplasma*-infected men rated the odour of a higher dose cat urine sample as more pleasant than the *Toxoplasma*-free men, while *Toxoplasma*-infected women rated it as less pleasant than *Toxoplasma*-free females. No significant effect of toxoplasmosis on the urine odour pleasantness scores was observed for other animal species, namely the horse, tiger, hyena, and dog.

The opposite reactions of infected male and female rodents to cat urine odour have not been reported yet. The phenomenon of fatal attraction to cat urine odour in infected mice or rats has been observed in both males and females. However, the experiments were conducted exclusively in either male rats [15]–[17] or female mice [15]–[17], [19] or the results were reported without indicating the sex of experimental animals [14]–[18]. In humans, the sex-specific opposite behavioural and physiological reactions to *Toxoplasma* infection are commonplace. It has been reported repeatedly that infected men have a decreased superego strength (Cattell's personality factor G), increased suspiciousness (factor L) and decreased affectothymia (factor A) while the opposite is true for these factors in *Toxoplasma*-infected women. In behavioural tests and observational studies, infected men expressed less and infected women more selfcontrol than *Toxoplasma*-free subjects [30], [31]. Infected male students also scored lower in clothes tidiness (they wore less clean, older and cheaper clothes) while infected female students scored higher in tidiness in comparison with uninfected female students [32], [33]. The behavioural differences between sexes were also observed during experimental games – the infected male students were less generous and infected women more generous (i.e. voluntarily returned more money to “investors”) during the Trust game [45]. The infected male students had higher while infected female students had lower levels of testosterone in saliva in comparison with their uninfected counterparts [46]. It might be indicative that in contrast to humans, the infected mice of both sexes had a decreased level of testosterone [47].

Lindová et. al. (2006) suggested so called stress hypothesis as a possible explanation of the opposite effects of latent toxoplasmosis on men and women personality and behaviour. This hypothesis is based on the assumption that latent toxoplasmosis, via deteriorated physical functioning, causes long-term subliminal stress. It is known that men and women cope differently with stress. In contrast to men, who seem to use more individualistic and antisocial (e.g. aggressive, hostile) forms of coping with stress, women are more likely to seek and provide social support, join with others, verbalize towards others or the self. A recent biological (evolutionary) theory similarly distinguishes between the male “fight-or-flight” response and the female “tend-and-befriend” reaction to stress following from the strong need of women to protect children and maintain social relationships. For detailed discussion of the theoretical background of the stress hypothesis, see [45]. It can be analogically speculated that infected men and women (in comparison with *Toxoplasma*-free subjects) could express opposite reaction on danger stimuli, e.g. a smell of predator, namely, the men could have increased and women decreased tendency to risk a contact with a predator.

We can only speculate on the reasons for the absence of any effects of toxoplasmosis on the scores attributed to the second feline species, the tiger, in our urine sample set. Both cat and tiger (in contrast with other species in our study) are definitive hosts of *Toxoplasma*. Moreover, the *Toxoplasma* bradyzoites had higher probability to enter the stomach of a tiger than that of a domestic cat in our recent evolutionary past. Presently, the urine component responsible for the fatal attraction phenomenon is not known. Based on our results, it can be suggested that felinine (2-amino-7-hydroxy-5,5-dimethyl-4-thiaheptanoic acid) might be implicated in the olfactory preference changes in *Toxoplasma*-infected intermediate hosts. This characteristic volatile component of the feline urine is involved in scent marking and, at natural temperature, is slowly released from its transporter proteins in urine for more than 30 days. Felinine is present only in the urine of small cats (Felinae subfamily) and not in that of large cats, tigers, lions, etc. (Pantherinae subfamily) [48]. It should be noted, however, that the effect of toxoplasmosis on olfactory preferences follows an inverted-U function - the effect was not observed when using either a high or very low amount of cat urine in the mice experiments [16]. In contrast to a relatively pleasant odour of low amounts of domestic cat urine, tiger urine odour was perceived as very unpleasant by most of our raters. Therefore, we could easily miss the fatal attraction peak for tiger urine because we had to use too small amounts of tiger urine for both the low and high concentration sample due to its strong ammonia smell.

We are aware of some particular limitations of the present study. There was a relatively low number of *Toxoplasma*-infected students among the study population. This number (fifteen men and nineteen women) was similar or higher than that of infected mice used in the previous animal studies; however, in various human behavioural studies, a higher number of infected subjects have always been used. The main reason was the substantial decrease in the prevalence of latent toxoplasmosis from 24% to about 15% among the students of the Faculty of Science of Charles University in the last decade. We tried to “enrich” our experimental set with *Toxoplasma*-infected individuals (our study is designed as a case control study rather than a cohort study); however, we had not enough infected students willing to participate voluntarily in our olfactory experiment in our file. The moderate number of infected students in our experiment could not lead to false positive results of statistical tests, e.g. to a detection of statistically significant but in fact non-existent effect of toxoplasmosis on the score attributed to the cat urine odour pleasantness. However, it could lead to false negative results, for example a missed effect of toxoplasmosis on urine odour pleasantness scores attributed to other animal species. A possible candidate for such a missed effect was the brown hyena which expressed a similar, but nonsignificant (p = 0.074) trend as the domestic cat.

The general aim of the study was explained in the informed consent. The study subjects thus knew that the focus was on possible influence of latent toxoplasmosis on olfactory sensitivity and preferences. Although the fatal attraction phenomenon was not referred to, some of the students may have been aware of it from e.g. some Czech textbooks, Wikipedia, mass media, etc.. However, in the rating form, the subjects were asked not only to score the intensity and pleasantness of particular odours but also to guess the nature of every particular sample as well as the aim of the whole experiment. Most of them guessed wrong – for example, cat urine odour was often mistaken for the smell of the sea, mushrooms or hospital. Only one student (out of 168) guessed right the specific aim of the study. However, none of the study subjects was aware of his/her toxoplasmosis status before or during the experiment and therefore, even hypothetical knowledge of the specific aim of the study could not result in systematic bias and consequent false positive results.

The observational studies can reveal existence of statistical association between two factors, e.g. between the toxoplasmosis and changed olfactory functions, however, they cannot determine causality. As one anonymous reviewer suggested, a possible explanation of the observed association is that individuals with past experience and familiarity with domestic cats might find the odour of their urine less unpleasant and also have had a higher chance of being exposed through their past contact with cats (i.e., the association is not caused by toxoplasma, but both infection and odour preferences are cause by past association with cats). However, this explanation seems to be less probable because: 1) the same association was observed also in the experiments with artificially infected animals 2) the odour of cat was less, not more, pleasant for the infected women than for uninfected controls. We used five (cats, hyenas, tigers) or three (horses, dogs) independent urine samples for each animal species to reduce the effect of pseudoreplications. To fully eliminate such an effect, it would be necessary to use the urine of a different animal for each rater, which would be technically difficult or even impossible for some animal species (there are probably not enough tigers in the Czech zoos for this design). We regularly rotated the urine samples in 17 experimental sessions and always focused on the similar frequency of *Toxoplasma* infected subjects in each section to block the possible effect of interindividual differences in the quality of urine samples from the same animal species. Due to the already mentioned effect of odour-specific hyposmia on the completeness of data, it would be difficult to eliminate the effect of the individual specimens during our analyses by using a more complex statistical models and more complex statistical techniques (Generalized Linear Mixed Models). Therefore, even in future studies, the effect of pseudoreplications cannot be fully avoided (as is usually the case, although rarely reflected or admitted in other studies).

Our results confirmed the existence of specific changes in the attractiveness of urine odour of a definitive host of *Toxoplasma* – the domestic cat, for latently infected individuals, in taxonomically unrelated host species, humans (Primates). In contrast to other studies dealing with this phenomenon, we searched for the effect of the same urine samples on olfactory preference in both male and female hosts, which enabled us to recognise the opposite directions of the toxoplasmosis-associated preference shift in males compared to females. Because the strength of the effect of toxoplasmosis on the olfactory preferences varies non-monotonically with the concentration of urine (and can be expressed as an inverted U function [16]), it probably could not be possible to reveal the toxoplasmosis-sex interaction in the isolated experiments performed separately with male and female rodents. In the view of our results, it seems to be important to repeat the standard animal arena experiments testing the phenomenon of fatal attraction with both males and females. Our study should be repeated in the future with the urine of more representatives of the Felininae and Pentherinae subfamilies. If our anecdotal observation concerning the difference between effects of toxoplasmosis on the urine odour pleasantness scores attributed to small and large cats were confirmed, then it could also provide a clue as to where and how to search for the component of urine that is responsible for the fatal attraction phenomenon [33], [49].

The possible absence of the effects of toxoplasmosis on the urine odour pleasantness score attributed to large cats would suggest that the amino acid felinine [48] could be responsible for the fatal attraction phenomenon. Our results also raise the possibility that the odour-specific threshold deficits observed in schizophrenia patients [43] could be in fact caused by increased prevalence of *Toxoplasma*-infected subjects in this population [35], [36] rather than by schizophrenia itself, which should be tested. Last but definitely not the least, the trend observed with the hyena urine sample suggests that this carnivore, and other representatives of the Feliformia suborder, should be studied for their possible role as definitive hosts in the life cycle of *Toxoplasma gondii*.

The change of olfactory functions in *Toxoplasma*-infected men and women in general population have probably only minor impacts on their life However, it might have an important impact on the progression of schizophrenia by contributing to symptoms of the disease such as olfactory hallucinations and paranoia. Therefore, the mechanism and nature of latent toxoplasmosis-associated changes in human olfactory functions deserve further attention in future studies.
